# Choriocapillaris Flow Deficits and Outer Retinal Biomarkers in Early Macular Telangiectasia Type 2: A Multimodal Imaging Study

**DOI:** 10.3390/biomedicines14102332

**Published:** 2026-10-14

**Authors:** Feliciana Menna, Alessandro Meduri, Laura De Luca, Antonio Baldascino, Enzo Maria Vingolo, Stefano Lupo

**Affiliations:** 1 Department of Medical-Surgical Sciences and Biotechnologies, U.O.C. Ophthalmology, Sapienza University of Rome, Via Firenze 1, 04019 Terracina, Italy; 2 Ophthalmology Clinic, Department of Biomedical Sciences, University of Messina, 98122 Messina, Italy; 3 Ophthalmology Department, Fondazione Policlinico Universitario A. Gemelli, IRCCS, 00168 Rome, Italy

**Keywords:** Macular Telangiectasia Type 2, OCT angiography, choriocapillaris, ellipsoid zone, Müller cells, retinal biomarkers, microperimetry, multimodal imaging

## Abstract

**Background:** Macular Telangiectasia Type 2 (MacTel Type 2) is a rare bilateral retinal disorder characterized by progressive neurodegeneration, Müller cell dysfunction, photoreceptor impairment, and secondary vascular remodeling. Although OCT and OCT angiography have improved the identification of structural and vascular abnormalities, the relationship between choriocapillaris impairment and early outer retinal degeneration remains incompletely defined. **Methods:** In this observational cross-sectional study, patients with early-stage MacTel Type 2 underwent multimodal imaging including spectral-domain OCT, OCT angiography, fundus autofluorescence, and microperimetry. Quantitative parameters included choriocapillaris flow deficit percentage, superficial and deep capillary plexus vessel density, ellipsoid zone disruption, central and parafoveal retinal thickness, hyporeflective intraretinal cavities, best corrected visual acuity, and mean retinal sensitivity. Analyses used linear mixed-effects models with a random intercept for patient to account for inter-eye correlation. **Results:** A cohort of 22 patients (40 eyes) with early non-neovascular MacTel Type 2 and 20 age-matched healthy control subjects (20 eyes) was analyzed. Mean age was 58.6 ± 7.9 years. Mean BCVA was 0.18 ± 0.11 logMAR, while mean retinal sensitivity was 24.1 ± 3.6 dB. Choriocapillaris flow deficits were increased in MacTel eyes compared with age-matched controls (18.7 ± 5.4% vs. 11.2 ± 3.8%; *p* < 0.001). Ellipsoid zone disruption was present in 55% of eyes, and greater EZ disruption length was significantly associated with reduced retinal sensitivity (standardized β = −0.61; *p* = 0.003). Choriocapillaris flow deficits were associated with greater ellipsoid zone disruption length (β = 0.58; *p* = 0.005), lower deep capillary plexus vessel density (β = −0.52; *p* = 0.013), and reduced retinal sensitivity (β = −0.49; *p* = 0.021). **Conclusions:** Early MacTel Type 2 was characterized by measurable OCTA-derived choriocapillaris flow impairment associated with outer retinal structural abnormalities and functional loss despite relatively preserved visual acuity. These cross-sectional associations do not establish temporal sequence or prognostic value; the utility of combined OCT/OCTA and microperimetric biomarkers for staging and monitoring requires prospective confirmation.

## 1. Introduction

Macular Telangiectasia Type 2 (MacTel Type 2) is a rare, bilateral, acquired retinal disorder characterized by progressive neurodegeneration, Müller cell dysfunction, photoreceptor degeneration, and secondary retinal vascular remodeling [1,2,3,4]. The disease typically manifests during the fifth to seventh decades of life and frequently remains undiagnosed in its early stages because central visual acuity is often preserved despite the presence of significant structural and functional abnormalities [5]. Patients commonly report metamorphopsia, reading difficulties, impaired contrast sensitivity, and paracentral scotomas, indicating that conventional visual acuity testing may underestimate the true functional impact of the disease [6].

Current evidence suggests that degeneration of Müller cells represents one of the earliest pathogenic events in MacTel Type 2. Loss of Müller cell support disrupts retinal homeostasis, resulting in photoreceptor degeneration, progressive ellipsoid zone (EZ) disruption, intraretinal cavitation, and subsequent remodeling of the retinal microvasculature [2,7]. Histopathological studies have demonstrated depletion of Müller cell markers together with abnormalities in macular pigment distribution, supporting the hypothesis that MacTel Type 2 is primarily a neurodegenerative disorder with secondary vascular alterations [7,8].

Spectral-domain optical coherence tomography (SD-OCT) has become the reference imaging modality for evaluating structural changes in MacTel Type 2. Typical findings include temporal parafoveal thinning, hyporeflective intraretinal cavities, external limiting membrane abnormalities, progressive ellipsoid zone disruption, and retinal atrophy [9,10,11]. Among these biomarkers, the extent of ellipsoid zone loss has consistently shown the strongest correlation with visual function and disease progression [11].

Optical coherence tomography angiography (OCTA) has substantially improved the understanding of retinal microvascular alterations in MacTel Type 2 by allowing non-invasive visualization of both retinal and choroidal circulation. Previous investigations have demonstrated progressive reductions in vessel density, particularly within the deep capillary plexus, together with telangiectatic vessels, right-angle venules, and enlargement of the parafoveal avascular zone [12,13,14,15]. More recently, increasing attention has focused on the choriocapillaris, as impaired choroidal perfusion may contribute to outer retinal degeneration through reduced metabolic support of the retinal pigment epithelium and photoreceptors [16,17,18]. However, quantitative choriocapillaris flow-deficit analysis has so far been developed and applied mainly in healthy eyes and in age-related macular degeneration [17,18], whereas OCTA studies of MacTel Type 2 have concentrated on the retinal capillary plexuses [12,13,14,15,16]. Studies that have quantified choriocapillaris flow deficits in the early, non-neovascular stage of MacTel Type 2 and related them, within the same eyes, to ellipsoid zone integrity and to fundus-controlled microperimetry remain scarce. Consequently, whether choriocapillaris flow deficits are already measurable at this stage, and how they relate to photoreceptor integrity and to localized retinal function in the same eyes, remains undefined.

Fundus-controlled microperimetry represents one of the most sensitive techniques for evaluating localized retinal function because retinal sensitivity closely reflects photoreceptor integrity and frequently declines before measurable reductions in best corrected visual acuity occur [19,20,21]. Integrating structural, vascular, and functional imaging may therefore provide a more comprehensive assessment of disease severity than any individual modality alone.

The present study was designed to address this specific gap. We hypothesized that, in early non-neovascular MacTel Type 2, OCTA-derived choriocapillaris flow deficits are already increased compared with healthy eyes and are associated, within the same eyes, with the extent of ellipsoid zone loss and with reduced microperimetric retinal sensitivity, even while best corrected visual acuity remains largely preserved. Accordingly, the primary objective was to quantify the choriocapillaris flow-deficit percentage in eyes with early non-neovascular MacTel Type 2 and to compare it with that of age-matched healthy eyes using linear mixed-effects models that account for the inclusion of both eyes of the same patient. The secondary objectives were (i) to test, in affected eyes, the associations of choriocapillaris flow deficits with ellipsoid zone disruption length, deep capillary plexus vessel density, and retinal sensitivity measured by fundus-controlled microperimetry, and (ii) to compare microperimetric retinal sensitivity with best corrected visual acuity as functional correlates of these early structural and vascular changes. The specific contribution of this work is therefore a cross-sectional, eye-matched quantification of the association between choriocapillaris perfusion, photoreceptor integrity, and retinal function at the earliest, non-neovascular stage of MacTel Type 2, in which visual acuity is still preserved; it does not address the temporal sequence, causality, or prognostic value of these changes, which require longitudinal data.

## 2. Materials and Methods

This observational cross-sectional study included clinical and imaging data collected during routine ophthalmic care at the Department of Ophthalmology of A. Fiorini Hospital, Terracina, Italy, between January 2024 and March 2026. The analysis of these data was approved by the competent Ethics Committee (Approval No. 657/2025, 1 December 2025), in accordance with the Declaration of Helsinki. Written informed consent was obtained from all participants.

Consecutive patients with a diagnosis of bilateral early-stage non-neovascular Macular Telangiectasia Type 2 examined during the study period were considered for inclusion. Eyes with evidence of subretinal neovascularization, diabetic retinopathy, retinal vein occlusion, glaucoma, high myopia exceeding 6 diopters, previous vitreoretinal surgery, significant media opacities, or any retinal disorder potentially affecting retinal sensitivity were excluded. Twenty-two patients corresponding to 40 eyes fulfilled the inclusion criteria. Both eyes were eligible in 18 patients, whereas in four patients only one eye fulfilled all predefined inclusion and image-quality criteria; therefore, the fellow eye was excluded from the analysis. Twenty age-matched healthy control subjects were recruited among individuals attending our ophthalmology clinic for routine examination. Control subjects had no history or clinical evidence of retinal or optic nerve disease and underwent the same imaging protocol. When both eyes fulfilled the eligibility criteria, one eye per participant was randomly selected for analysis.

Each participant underwent a complete ophthalmologic examination including best corrected visual acuity (BCVA), slit-lamp biomicroscopy, Goldmann applanation tonometry, dilated fundus examination, fundus autofluorescence (FAF), spectral-domain optical coherence tomography (SD-OCT), optical coherence tomography angiography (OCTA), and fundus-controlled microperimetry.

Structural OCT analysis included central retinal thickness, parafoveal retinal thickness, ellipsoid zone disruption length, external limiting membrane abnormalities, hyporeflective intraretinal cavities, and hyperreflective foci. Ellipsoid zone disruption was manually measured on the horizontal B-scan passing through the foveal center by two independent masked retinal specialists, and the mean value was used for statistical analysis.

Structural and angiographic imaging was performed using the Xephilio OCT-A1 (Canon Medical Systems, Otawara, Japan), a spectral-domain optical coherence tomography system operating at 70,000 A-scans/s with a central wavelength of approximately 855 nm. OCTA images were acquired using a 6 × 6 mm scan pattern centered on the fovea, with two repeated B-scans acquired at each scan location. Automatic segmentation of the superficial capillary plexus (SCP), deep capillary plexus (DCP), and choriocapillaris (CC) was carefully reviewed in all examinations and manually corrected whenever segmentation errors were identified by a trained operator (F.M.) using the segmentation-editing tool of the device software.

For choriocapillaris analysis, the en-face CC slab generated by the Angio Expert software (Canon Medical Systems, Otawara, Japan; https://eu.medical.canon/products/eye-care/xephilio-oct-a1/octa-and-intelligent-denoise/, accessed on 11 October 2026) was analyzed over the entire 6 × 6 mm fovea-centered scan area. The slab was defined relative to the automatically segmented Bruch’s membrane using the manufacturer-provided choriocapillaris preset. Automatic segmentation was reviewed on individual B-scans and manually corrected when necessary before regeneration of the en-face slab. Projection artifacts from the overlying retinal circulation were reduced using the projection-artifact removal algorithm implemented in the Canon Angio Expert software. The entire scan area, including the central foveal region, was included in the CC FD calculation, with no exclusion of the central region. The same slab preset and analysis configuration were used for all MacTel and control eyes. Choriocapillaris flow-deficit measurements were obtained by the same operator (F.M.) using the proprietary Canon analysis procedure, in which choriocapillaris flow deficits are defined as areas lacking detectable OCTA flow signal; CC flow deficit (CC FD) percentage was calculated as the total area classified as flow deficit divided by the total analyzed choriocapillaris area, multiplied by 100. The numerical internal threshold parameters were not modified by the investigators and were not available as user-exportable parameters in the software configuration used for the present analysis. Accordingly, the reported CC FD values should be interpreted as platform-specific OCTA flow-signal deficit measurements and may not be directly interchangeable with values obtained using different devices or post-processing algorithms.

No dedicated signal-attenuation compensation was applied during post-processing. Consequently, the CC FD values reported in this study should be interpreted as OCTA flow-signal deficits, which may reflect both true reductions in choriocapillaris perfusion and reduced signal transmission through the overlying outer retina and retinal pigment epithelium, particularly in areas of ellipsoid zone disruption, hyperreflective foci, or RPE alteration; this caveat is taken into account in the interpretation of the results. Only OCTA acquisitions with an image-quality score ≥ 7 were included in the quantitative analysis. Images showing significant motion artifacts, uncorrectable segmentation errors, or insufficient signal quality were excluded; these image-quality criteria were applied at the eye-selection stage described above, and all 40 included MacTel eyes and 20 control eyes fulfilled them.

OCTA abnormalities were primarily assessed quantitatively using vessel-density and flow-deficit metrics rather than by subjective grading of their visual conspicuity on en-face images.

FAF imaging was acquired using the Canon CX-1 Digital Mydriatic Retinal Camera (Canon Inc., Tokyo, Japan) in dedicated FAF mode, using an excitation wavelength range of approximately 530–580 nm. FAF images were evaluated for abnormalities of macular pigment distribution, temporal parafoveal hyperautofluorescence, and retinal pigment epithelium alterations.

Retinal function was assessed using the MP-3 Microperimeter (Nidek Co., Ltd., Gamagori, Japan). Microperimetry was performed with a standardized central 10° testing strategy using a Goldmann III stimulus, a 4-2 staircase algorithm, a background luminance of 31.4 apostilbs, and a stimulus duration of 200 ms. The instrument incorporates real-time eye tracking and automatic fundus registration, allowing accurate point-to-point correlation between retinal sensitivity and structural imaging findings. Mean retinal sensitivity, expressed in decibels (dB), was considered the primary functional outcome measure.

The imaging protocol was designed for standardized quantitative and qualitative assessment of predefined structural and vascular biomarkers across the entire cohort rather than for illustrative case-based analysis.

Statistical analyses were performed using IBM SPSS Statistics, version 31.0 (IBM Corp., Armonk, NY, USA). Normality of continuous variables was assessed using the Shapiro–Wilk test, with the significance level (α) set at 0.05; the test statistic (W) and the corresponding *p* value obtained for each variable in each group are reported in Table 1. As no variable showed a significant departure from normality (all *p* > 0.05), continuous variables were expressed as mean ± standard deviation. Because both eyes were included for 18 of the 22 patients with MacTel Type 2, eyes from the same participant could not be regarded as independent observations, and all eye-level analyses accounted for inter-eye correlation. Between-group comparisons of eye-level variables (BCVA, retinal sensitivity, central retinal thickness, SCP and DCP vessel density, and CC FD) were performed using linear mixed-effects models with group (MacTel vs. control) as a fixed effect and a random intercept for participant, estimated by restricted maximum likelihood; results are reported as model-estimated mean differences with 95% confidence intervals (CI). Participant-level variables (age and sex) were compared using Student’s *t*-test and Fisher’s exact test, respectively. Associations between structural, vascular, and functional parameters within MacTel eyes were likewise assessed using linear mixed-effects models with a random intercept for participant, in which one parameter was entered as the dependent variable and the other as a fixed-effect covariate. Both variables were standardized (z-scores) before modeling, so that the resulting fixed-effect coefficients (standardized β) are expressed on a scale comparable to that of a correlation coefficient. For transparency, the unadjusted Pearson correlation coefficients obtained when all eyes were treated as independent observations are also reported alongside the mixed-model estimates. Statistical significance was defined as *p* < 0.05.

## 3. Results

A total of 22 patients (40 eyes) with early-stage non-neovascular Macular Telangiectasia Type 2 and 20 age-matched healthy control subjects (20 eyes) were included in the study. Baseline demographic and clinical characteristics are summarized in Table 2. Model-estimated between-group mean differences with 95% confidence intervals, derived from the linear mixed-effects models, are reported in Table 2 for all eye-level variables. The mean age of patients with MacTel Type 2 was 58.6 ± 7.9 years, and 13 patients (59.1%) were female. All included eyes completed the multimodal imaging protocol and were included in the final analysis. Both eyes were included for 18 patients and one eye for the remaining 4 patients; the resulting inter-eye correlation was accounted for in all eye-level analyses, as described above.

The results of the Shapiro–Wilk normality test for each continuous variable are reported in Table 1. At the pre-specified significance level of α = 0.05, no variable showed a significant departure from normality in either group (all *p* > 0.05), supporting the use of parametric statistics throughout the analysis.

Mean BCVA was 0.18 ± 0.11 logMAR, indicating relatively preserved central vision, whereas mean retinal sensitivity measured by fundus-controlled microperimetry was 24.1 ± 3.6 dB, demonstrating the presence of localized functional impairment despite limited reduction in visual acuity.

Structural SD-OCT analysis revealed EZ disruption in 22 of the 40 eyes (55.0%). The mean EZ disruption length was 612 ± 328 μm. Interobserver agreement for EZ disruption length was assessed using the intraclass correlation coefficient (ICC), based on a two-way random-effects model with absolute agreement. Interobserver agreement for EZ disruption length measurements was excellent (ICC = 0.92; 95% CI, 0.86–0.96).

Hyporeflective intraretinal cavities were identified in 28 eyes (70.0%), predominantly within the temporal parafoveal region, while hyperreflective foci were observed in 17 eyes (42.5%). Mean central retinal thickness was 246.8 ± 18.7 μm and mean parafoveal retinal thickness was 287.4 ± 21.6 μm.

**Figure 1:**
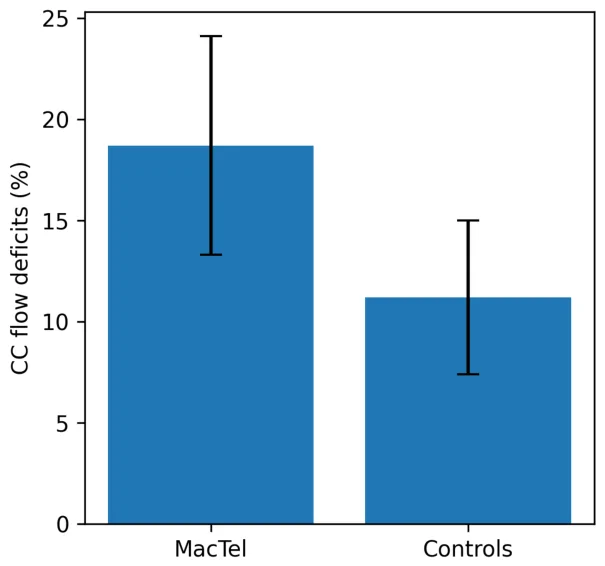
Comparison of choriocapillaris flow deficit percentage between eyes with early Macular Telangiectasia Type 2 and healthy controls. MacTel eyes showed significantly greater OCTA-derived choriocapillaris flow-deficit measurements than healthy controls (18.7 ± 5.4% vs. 11.2 ± 3.8%; *p* < 0.001, linear mixed-effects model accounting for inter-eye correlation). Bars represent mean values and error bars the standard deviation.

Quantitative OCTA analysis demonstrated significantly greater choriocapillaris flow-deficit measurements in MacTel eyes compared with healthy controls (18.7 ± 5.4% vs. 11.2 ± 3.8%; mixed-model estimated mean difference 7.5%, 95% CI 4.6 to 10.4; *p* < 0.001), as illustrated in Figure 1.

**Figure 2:**
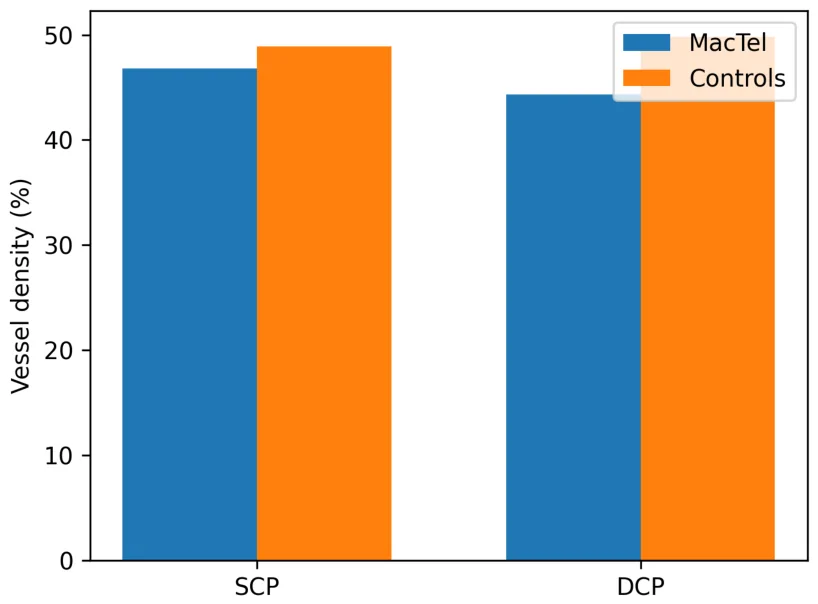
Comparison of superficial and deep capillary plexus vessel density between MacTel Type 2 eyes and healthy controls. Deep capillary plexus vessel density demonstrated the greatest reduction in affected eyes, whereas superficial plexus changes were less pronounced. Bars represent mean values; *p* values from linear mixed-effects models accounting for inter-eye correlation are reported in Table 2.

Deep capillary plexus vessel density was markedly reduced in MacTel eyes compared with controls (44.3 ± 4.9% vs. 49.8 ± 3.7%; estimated difference −5.5%, 95% CI −8.2 to −2.8; *p* < 0.001), whereas the difference in superficial capillary plexus vessel density was smaller and did not reach statistical significance (46.8 ± 3.8% vs. 48.9 ± 3.5%; estimated difference −2.1%, 95% CI −4.4 to 0.2; *p* = 0.070), as shown in Figure 2.

**Figure 3:**
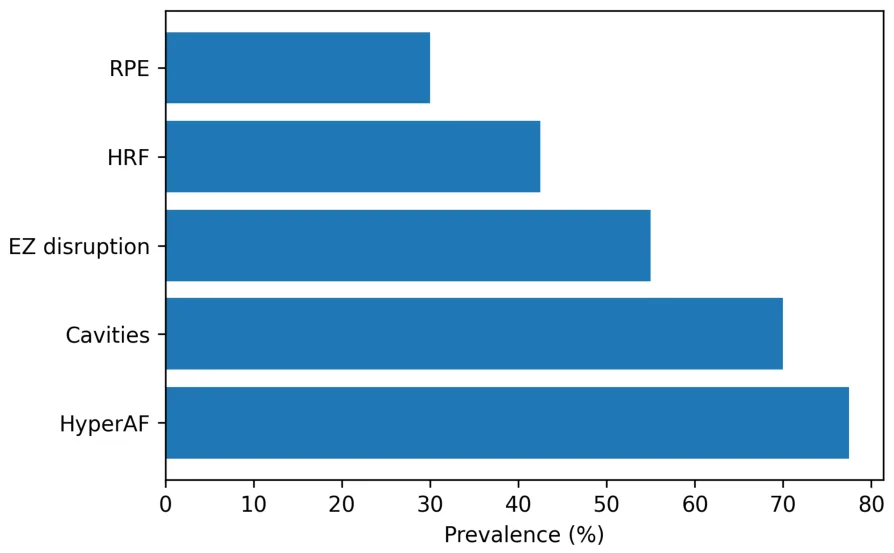
Prevalence of major structural retinal biomarkers detected by spectral-domain optical coherence tomography (SD-OCT) using the Canon Xephilio OCT-A1 and fundus autofluorescence in early Macular Telangiectasia Type 2 (n = 40 eyes). RPE, retinal pigment epithelium alterations; HRF, hyperreflective foci; EZ, ellipsoid zone disruption; HyperAF, hyperautofluorescence. Hyperautofluorescence and intraretinal cavitations represented the most frequent abnormalities, followed by EZ disruption and HRF.

Fundus autofluorescence demonstrated temporal parafoveal hyperautofluorescence in 31 eyes (77.5%), while abnormalities of macular pigment distribution were observed in all affected eyes. Areas of retinal pigment epithelium alteration were present in 12 eyes (30.0%). The prevalence of the principal structural retinal biomarkers identified by SD-OCT and fundus autofluorescence is shown in Figure 3.

In linear mixed-effects models accounting for inter-eye correlation, choriocapillaris flow deficits were significantly associated with ellipsoid zone disruption length (standardized β = 0.58, 95% CI 0.21 to 0.80; *p* = 0.005). Greater choriocapillaris flow deficits were also associated with lower deep capillary plexus vessel density (β = −0.52, 95% CI −0.77 to −0.13; *p* = 0.013) and with reduced retinal sensitivity (β = −0.49, 95% CI −0.76 to −0.09; *p* = 0.021). The strongest structure–function association was observed between ellipsoid zone disruption length and retinal sensitivity (β = −0.61, 95% CI −0.82 to −0.25; *p* = 0.003), indicating that greater photoreceptor loss was closely associated with localized functional deterioration. The mixed-model point estimates were essentially unchanged compared with the unadjusted Pearson coefficients obtained when eyes were treated as independent observations, whereas confidence intervals were wider and *p* values larger, as expected when inter-eye correlation is taken into account (Table 3).

In contrast, BCVA showed only weak associations with choriocapillaris flow deficits (β = 0.24, 95% CI −0.20 to 0.60; *p* = 0.282) and ellipsoid zone disruption length (β = 0.29, 95% CI −0.15 to 0.63; *p* = 0.190), suggesting that conventional visual acuity is relatively insensitive to early structural and vascular alterations in MacTel Type 2. Mixed-model estimates for all pairwise associations, together with the corresponding unadjusted Pearson coefficients, are reported in Table 3.

Overall, the combined multimodal imaging findings indicate that OCTA-derived choriocapillaris flow deficits are associated with outer retinal degeneration and localized functional loss in the early stages of MacTel Type 2, in eyes in which central visual acuity is still relatively preserved. These cross-sectional associations do not, by themselves, indicate which change occurs first.

## 4. Discussion

The present study demonstrates that early non-neovascular MacTel Type 2 is characterized by increased OCTA-derived choriocapillaris flow deficits that are associated with outer retinal structural degeneration and localized functional loss despite relatively preserved best corrected visual acuity, and that these associations persist after accounting for the inclusion of both eyes of the same patient. These findings indicate that choriocapillaris flow abnormalities are detectable in early disease and are associated with photoreceptor structural changes.

Previous OCTA investigations have primarily focused on retinal vascular remodeling, particularly reductions in deep capillary plexus vessel density and the presence of parafoveal telangiectatic vessels [12,13,14,15]. Our findings extend current knowledge by demonstrating that quantitative choriocapillaris flow deficits are significantly associated with both ellipsoid zone disruption and retinal sensitivity loss, indicating an association between alterations of the choroidal microcirculation and early outer retinal degeneration. To our knowledge, few studies have examined choriocapillaris flow deficits, photoreceptor integrity, and fundus-controlled microperimetry together in the same eyes in the early non-neovascular stage of the disease; this eye-matched, multimodal characterization of the earliest stage, in which visual acuity is still largely preserved, is the specific contribution of the present work.

The observed relationship between choriocapillaris flow deficits and ellipsoid zone disruption is biologically plausible because the choriocapillaris provides the principal vascular supply to the retinal pigment epithelium and photoreceptor outer segments. From a biological perspective, impaired choroidal perfusion could potentially compromise photoreceptor metabolism [16,17,18]; however, the cross-sectional nature of the present study does not allow the direction or causality of this relationship to be established. These findings further support the concept that neurodegeneration and vascular remodeling are closely interconnected during the evolution of MacTel Type 2. However, the association between CC FD and EZ disruption should be interpreted cautiously. Alterations of the outer retina and RPE, including EZ loss, RPE abnormalities, hyperreflective foci, and intraretinal cavitations, may modify OCT signal transmission to the choriocapillaris and potentially influence flow-deficit measurements. Therefore, part of the observed association between greater CC FD and EZ disruption may reflect signal-attenuation effects rather than true perfusion impairment alone. Accordingly, the present cross-sectional findings demonstrate an association but cannot establish a causal relationship between choriocapillaris impairment and photoreceptor degeneration.

An important observation of the present study is that retinal sensitivity measured by fundus-controlled microperimetry demonstrated stronger associations with structural biomarkers than conventional visual acuity. Although most patients maintained relatively good BCVA, localized reductions in retinal sensitivity closely reflected both ellipsoid zone disruption and increasing choriocapillaris flow deficits. These results are consistent with previous reports showing that microperimetry is considerably more sensitive than visual acuity for detecting early functional impairment in MacTel Type 2 [19,20,21].

The multimodal imaging approach adopted in this study allowed simultaneous evaluation of structural, vascular, and functional changes within the same examination. Combining SD-OCT, OCT angiography, fundus autofluorescence, and fundus-controlled microperimetry provides complementary biomarkers that characterize early disease involvement more completely than any single modality. Whether these biomarkers can improve disease staging, serve for longitudinal monitoring, or act as outcome measures in therapeutic trials targeting early neurodegeneration cannot be inferred from cross-sectional data and will require prospective longitudinal validation.

Several limitations should be acknowledged. First, the relatively small sample size reflects the rarity of MacTel Type 2 and may limit the generalizability of the findings. No a priori sample size calculation was performed because of the exploratory nature of the study and the limited availability of patients with early-stage disease. Second, both eyes were included for 18 of the 22 patients. Inter-eye correlation was accounted for by using linear mixed-effects models with a random intercept for participant in all eye-level analyses; nevertheless, with only 22 clusters the between-participant variance may be imprecisely estimated, and the results should be interpreted with this in mind. Third, choriocapillaris flow-deficit measurements may have been influenced by signal attenuation related to outer retinal and retinal pigment epithelium abnormalities, particularly in areas of greater ellipsoid zone disruption. As no dedicated signal-attenuation compensation was applied, the possibility that part of the observed association between CC FD and outer retinal damage reflects imaging-related effects rather than true perfusion abnormalities cannot be completely excluded. In addition, CC flow-deficit quantification relied on a manufacturer-specific proprietary analysis configuration; therefore, absolute CC FD values may not be directly comparable with measurements obtained using other OCTA platforms or independently implemented thresholding algorithms. Furthermore, although FAF abnormalities were frequently observed, particularly temporal parafoveal hyperautofluorescence, the present study was not designed to quantitatively or topographically assess the relationship between individual FAF patterns and functional outcomes. Therefore, no specific conclusions can be drawn regarding the association of FAF abnormalities with BCVA or retinal sensitivity. Finally, the cross-sectional design precludes determination of the temporal or causal relationship between choriocapillaris abnormalities and photoreceptor degeneration, and does not allow any inference about whether the biomarkers examined here can track disease progression or predict functional decline over time. Accordingly, the observed associations should not be interpreted as evidence of a causal relationship between choriocapillaris impairment and outer retinal degeneration. Prospective longitudinal studies with larger cohorts, attenuation-compensated choriocapillaris imaging, and quantitative FAF analysis spatially co-registered with fundus-controlled microperimetry are required to validate these findings and determine whether baseline choriocapillaris flow deficits predict subsequent enlargement of ellipsoid zone defects and progressive functional deterioration.

## 5. Conclusions

Early non-neovascular MacTel Type 2 is characterized by increased OCTA-derived choriocapillaris flow-deficit measurements that are associated with outer retinal structural abnormalities and localized functional loss despite relatively preserved central visual acuity, and these associations persist after accounting for inter-eye correlation. Quantitative assessment of choriocapillaris flow deficits, together with evaluation of ellipsoid zone integrity and retinal sensitivity, provides complementary biomarkers for characterizing early disease involvement. Because of the cross-sectional design and the absence of signal-attenuation compensation, these findings establish associations only: they do not indicate which change occurs first, nor whether these biomarkers can be used to stage or monitor the disease over time. Prospective longitudinal studies with larger patient cohorts are warranted to determine the prognostic value of these imaging biomarkers and to clarify the role of choriocapillaris dysfunction in the pathophysiology of MacTel Type 2.

## Figures and Tables

**Table 1:** Shapiro–Wilk normality test results for continuous variables in the MacTel Type 2 and control groups.

Variable	Group	*n*	Shapiro–Wilk W	*p* Value
Age (years)	MacTel	22	0.978	0.875
Age (years)	Controls	20	0.935	0.195
BCVA (logMAR)	MacTel	40	0.982	0.748
BCVA (logMAR)	Controls	20	0.969	0.732
Retinal sensitivity (dB)	MacTel	40	0.945	0.051
Retinal sensitivity (dB)	Controls	20	0.961	0.563
CRT (µm)	MacTel	40	0.979	0.658
CRT (µm)	Controls	20	0.960	0.550
SCP vessel density (%)	MacTel	40	0.982	0.766
SCP vessel density (%)	Controls	20	0.932	0.172
DCP vessel density (%)	MacTel	40	0.985	0.865
DCP vessel density (%)	Controls	20	0.979	0.921
CC flow deficit (%)	MacTel	40	0.949	0.068
CC flow deficit (%)	Controls	20	0.976	0.873

W, Shapiro–Wilk test statistic; *n*, number of observations. The significance level was set at α = 0.05; *p* values above this threshold indicate no significant departure from normality. Age was tested at the participant level; all other variables were tested at the eye level. Retinal sensitivity (*p* = 0.051) and CC flow deficit (*p* = 0.068) in the MacTel group approached but did not reach the threshold; given the sample size and the robustness of linear mixed-effects models to mild deviations from normality, parametric modeling was retained for all variables. BCVA, best corrected visual acuity; CRT, central retinal thickness; SCP, superficial capillary plexus; DCP, deep capillary plexus; CC, choriocapillaris.

**Table 2:** Baseline demographic and clinical characteristics.

Variable	MacTel (22 Patients, 40 Eyes)	Controls (20 Subjects, 20 Eyes)	Estimated Mean Difference (95% CI)	*p* Value
Age (years)	58.6 ± 7.9	57.9 ± 6.8	—	0.71
Female (%)	59.1	55.0	—	0.79
BCVA (logMAR)	0.18 ± 0.11	0.02 ± 0.04	0.16 (0.10 to 0.22)	<0.001
Retinal sensitivity (dB)	24.1 ± 3.6	28.4 ± 1.9	−4.3 (−6.2 to −2.4)	<0.001
CRT (µm)	246.8 ± 18.7	253.9 ± 15.8	−7.1 (−17.8 to 3.6)	0.190
SCP vessel density (%)	46.8 ± 3.8	48.9 ± 3.5	−2.1 (−4.4 to 0.2)	0.070
DCP vessel density (%)	44.3 ± 4.9	49.8 ± 3.7	−5.5 (−8.2 to −2.8)	<0.001
CC flow deficit (%)	18.7 ± 5.4	11.2 ± 3.8	7.5 (4.6 to 10.4)	<0.001

Data are presented as mean ± standard deviation or percentage, as appropriate. Normality of continuous variables was assessed using the Shapiro–Wilk test. Age was compared using Student’s *t*-test and sex using Fisher’s exact test at the participant level; ophthalmic and imaging variables were compared at the eye level using linear mixed-effects models with group as a fixed effect and a random intercept for participant, to account for the inclusion of both eyes of the same patient. Age and sex are reported at the participant level; ophthalmic and imaging variables are reported at the eye level. Estimated mean differences are the fixed-effect estimates for group (MacTel minus controls) from these models, with their 95% confidence intervals; negative values indicate lower values in MacTel eyes. —, not applicable (participant-level variables compared with Student’s *t*-test or Fisher’s exact test). BCVA, best corrected visual acuity; CRT, central retinal thickness; SCP, superficial capillary plexus; DCP, deep capillary plexus; CC, choriocapillaris; CI, confidence interval.

**Table 3:** Associations between structural, vascular, and functional parameters in early MacTel Type 2 (40 eyes of 22 patients): linear mixed-effects model estimates accounting for inter-eye correlation, with unadjusted Pearson correlation coefficients for comparison.

Association	Standardized β (95% CI)	*p* Value	Unadjusted Pearson r
CC FD—EZ disruption length	0.58 (0.21 to 0.80)	0.005	0.58
CC FD—Retinal sensitivity	−0.49 (−0.76 to −0.09)	0.021	−0.49
CC FD—DCP vessel density	−0.52 (−0.77 to −0.13)	0.013	−0.52
CC FD—BCVA	0.24 (−0.20 to 0.60)	0.282	0.24
EZ disruption length—Retinal sensitivity	−0.61 (−0.82 to −0.25)	0.003	−0.61
EZ disruption length—DCP vessel density	−0.39 (−0.70 to 0.04)	0.073	−0.39
EZ disruption length—BCVA	0.29 (−0.15 to 0.63)	0.190	0.29
Retinal sensitivity—DCP vessel density	0.42 (0.00 to 0.72)	0.052	0.42
Retinal sensitivity—BCVA	−0.33 (−0.66 to 0.11)	0.134	−0.33
DCP vessel density—BCVA	−0.20 (−0.57 to 0.24)	0.372	−0.20

Standardized β, fixed-effect coefficient from a linear mixed-effects model with a random intercept for participant, after z-score standardization of both variables (interpretable on the scale of a correlation coefficient); CI, confidence interval. Unadjusted Pearson r values are those obtained when all 40 eyes were treated as independent observations and are reported for comparison only. CC FD, choriocapillaris flow deficit; EZ, ellipsoid zone disruption length; BCVA, best corrected visual acuity (logMAR); DCP, deep capillary plexus vessel density.

## Data Availability

The original contributions presented in this study are included in the article. Further inquiries can be directed to the corresponding author.

## Abbreviations

The following abbreviations are used in this manuscript:

BCVA	Best Corrected Visual Acuity
CC	Choriocapillaris
DCP	Deep Capillary Plexus
EZ	Ellipsoid Zone
FAF	Fundus Autofluorescence
FD	Flow Deficit
MacTel	Macular Telangiectasia
OCT	Optical Coherence Tomography
OCTA	Optical Coherence Tomography Angiography
RPE	Retinal Pigment Epithelium
RS	Retinal Sensitivity
SCP	Superficial Capillary Plexus
